# Vibrational courtship disruption of *Nilaparvata lugens* using artificial disruptive signals

**DOI:** 10.3389/fpls.2022.897475

**Published:** 2022-07-22

**Authors:** Zelin Feng, Qi Wei, Zhongru Ye, Baojun Yang, Yufan Gao, Jun Lv, Yanyun Dai, Jia Bao, Qing Yao

**Affiliations:** ^1^School of Information Science and Technology, Zhejiang Sci-Tech University, Hangzhou, China; ^2^State Key Laboratory of Rice Biology, China National Rice Research Institute, Hangzhou, China

**Keywords:** *Nilaparvata lugens*, courtship signals, disruptive signals, courtship disruption, behavior response

## Abstract

The brown planthopper (BPH), *Nilaparvata lugens* (Stål; Hemiptera: Delphacidae) is a piercing-sucking pest that causes serious damage to rice plants by sucking the phloem sap from the plants and transmitting viruses. During courtship, the BPH vibrates its abdomen to produce signals that are transmitted to rice plants through its legs. Male BPHs search, locate, and mate with female BPHs after they exchange courtship signals with each other. Currently, spraying chemical pesticides is still the primary method for controlling BPH populations in paddy fields, although this approach has led to severe environmental pollution. A physical control method based on BPH courtship disruption to reduce the mating rate is a promising strategy for cutting environmental pollution. To acquire effective courtship disruptive signals, we developed a vibration signal recording, monitoring, and playback system for BPHs. Using this system, BPH courtship signals and male competition signals were collected and analyzed to obtain their frequency spectra. Results show that the mean main vibration frequency of female courtship signals is 234 Hz and the mean pulse rate is 23 Hz. The mean main vibration and pulse frequencies of the male courtship signals are 255 Hz and 82 Hz, respectively. Besides, the mean main vibration frequency of the male competition signal is 281 Hz. Seven different forms and frequencies of artificial signals were played back to male BPHs, then the courtship and behavioral responses of male BPHs were analyzed. Results indicate that a pure tone of 225 Hz prevents the males from recognizing female courtship signals. The male reply rate fell from 95.6 to 33.3% and the mean reply delay time increased from 5.3 s to 9.1 s. The reply rates of the other six artificial signals ranged from 42.9 to 83.7%, and the mean reply delays were between 5.0 s and 9.3 s. Therefore, the courtship behavior of BPHs can be disrupted by using specific artificial disruptive signals.

## Introduction

**Figure 1:**
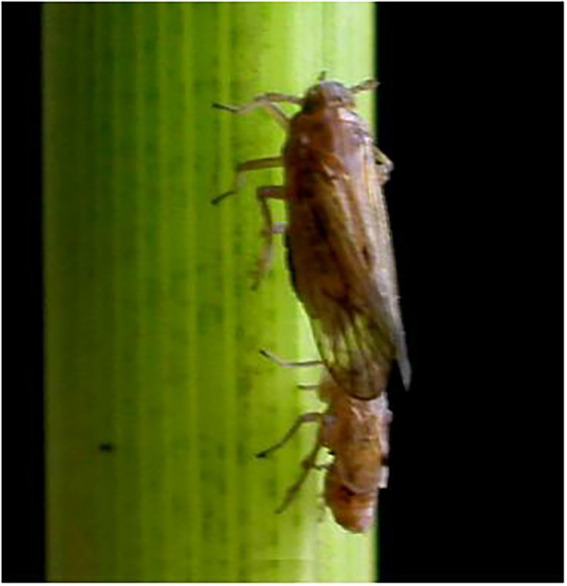
*Nilaparvata lugens* (adult).

The brown planthopper (BPH), *Nilaparvata lugens* (Stål; Hemiptera: Delphacidae), is an agricultural pest that feeds on rice sap using piercing-sucking mouthparts (Figure 1). BPH outbreaks lead to widespread plant death, which directly affects rice yield and quality (Dyck and Thomas, 1979). At present, BPH control mainly relies on chemical pesticides (Wu, 2018), but the extensive use of pesticides often leads to several issues including pesticide residue, death of natural enemies, pest resistance, and environmental pollution (Matsumura and Sanada-Morimura, 2010). Therefore, the exploration of new, efficient, and environmentally-friendly BPH physical control technologies to replace chemical pesticides has become the focus of much research in recent years.

Ossiannilsson (1949) demonstrated that most groups of Auchenorrhyncha generate low-intensity vibration signals and use them for communication. Ichikawa et al. (1975) recorded the courtship signals of three species of rice planthoppers, including BPH. The BPH vibration signal cannot be heard by the human ear and can only be gathered using specialized devices (Zhang and Chen, 1987b). Zhang and Chen (1991) developed a BPH vibration signal recording device for BPH vibration signals with a pickup, preamplifier, power amplifier, and recorder. The identified vibration signals of BPHs mainly occur during courtship and mating (Zhang and Chen, 1987a; Saxena et al., 1993), and the vibration signals of BPHs have three roles. These roles include the communication and identification of male and female individuals, stimulation of sexual arousal, and guidance for males in search of females (Zhang, 1997). On the same or neighboring rice plants, BPHs use vibrating courtship signals to contact the opposite sex. Females generally stay in one place, while males call and search according to the response signals (Zhang, 1997). At the same time, BPH males are capable of emitting vibration signals related to reproductive competition, which disturb the other males and reduce their mating rate (Zhang et al., 1991). Since the courtship vibration signals of BPHs are highly specific and stable (Cokl and Virant-Doberlet, 2003), new pest control technologies can be utilized to emit disruptive signals that upset the courtship communication of the target pest (Eriksson et al., 2012; Polajnar et al., 2016).

Mazzoni et al. (2009) used artificially simulated competition signals to disrupt the male and female mating behavior of *Scaphoideus titanus* and proposed a new vibration control technology for leafhoppers. This technology used a shaker device to generate the selected disruptive signal and transmit the signal to grape leaves through trellising wires. The amplitude of the signal was 7.5 mm at the source and dropped to 0.1 mm at a distance of 10 m from the sound source. The mating inhibition rate of the male/female leafhoppers was about 90% and there was no negative impact on the natural enemies of the leafhoppers in the vineyard (Polajnar et al., 2016). Fu et al. (1997, 1999) confirmed that the competition signal of males significantly reduced the mating rate of BPHs (41.0%) and had a significant inhibitory effect during reproduction.

Besides the male competition signal, it has yet to be established whether other forms of signals could disrupt BPH courtship and mating behavior more effectively. In this paper, we developed a system for collecting, monitoring, and playing back BPH vibration signals. Artificial digital signals of different frequencies and forms were generated using Python and Adobe Audition according to the sensitive frequencies of BPH courtship calls and male competition calls. The playback and monitoring experiments were conducted to determine the most effective courtship disruptive signals.

## Materials and methods

### BPH rearing

All tested BPH subjects were obtained from the insect rearing room of the China National Rice Research Institute. The BPHs were reared on fresh rice seedlings at 26 ± 1°C and 70 ± 10% humidity under a 16/8 h light/dark photoperiod. Newly-emerged virgin BPHs (<24 h) were transferred to separate cages to avoid mating.

### Development of BPH vibration signal recording, monitoring, and playback system

**Figure 2:**
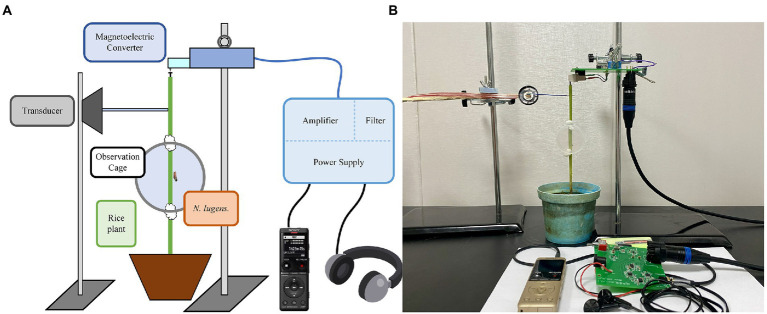
Vibration signal recording, monitoring, and playback system of BPH: **(A)** System framework diagram; **(B)** Physical image of the system.

To collect, monitor, and play back the vibration signals of BPHs, we developed a novel system with magnetoelectric converters, low-noise amplifier circuits, transducers, and other components. The setup is illustrated in Figure 2.

The magnetoelectric converter (model AT-3600 L) consists of a magnet, coil, and stylus. It converts the weak BPH vibration signal into a current output. The low-noise amplifier circuit utilizes the Texas Instruments bipolar high-performance audio operational amplifier (OPA1612), which has excellent noise performance and ultra-low distortion. To filter out noise from the BPH vibration signal and the signals in the non-target frequency band, we designed a Chebyshev sixth-order active band-pass filter. The system is powered by a lithium battery and the DC power circuit uses two integrated circuit chips: TPS563201 and LM27762. These devices support such functions as soft start, current limit, and thermal protection, as well as other functions. This power circuit has low noise and high-efficiency characteristics, and the 4,800 mah battery can be used continuously for more than 5 days. The transducer incorporates a 0.25 W flat-panel-driven full-frequency speaker. The signal collected through playback has no obvious distortion and only part of the frequency component is lost due to the mechanical filtering characteristics of rice plants. The system integrates power supply, amplification, and filter circuits with single-ended shielding and an optimized printed circuit board design to reduce noise during recording and monitoring.

### Recording of BPH courtship vibration signals

A healthy rice plant was planted in a small pot and the stems and leaves of the rice plant above 15 cm were removed. The BPH test subject was placed in a circular observation box that was positioned about 10 cm above the rice stem base. At the top of the main rice stem, an iron nail was inserted as the contact point of the magnetoelectric converter. The converter of the system was attached to an iron stand with an adjustable shaft. Additionally, the position of the counterweight was modified so that the converter rested lightly on the contact surface directly above the nail. The output of the system was connected to a recording pen for recording and to headphones for monitoring. In this study, a total of 30 male BPHs and 30 female BPHs were used to record signals.

### Generation of artificial disruptive signals

To disrupt the recognition of BPH courtship signals and male localization, we generated seven distinct disruptive signals of three different types. From the spectrum of the BPH courtship vibration signal in Section 3.1, the frequency of the female courtship signal is significantly in the range of 150 Hz to 300 Hz. Therefore, we selected the frequencies of 150 Hz, 225 Hz, and 300 Hz as the basis for the generation of artificial disruptive signals. Type 1 was the pure tone (PT) of different frequencies within the range of the female courtship signal. There were three kinds of PTs with frequencies of 150 Hz, 225 Hz, and 300 Hz. Type 2 was the continuous pulse signal (CPS), which had the same form as the female courtship signal. The three kinds of CPS had a constant pulse rate (PR) of 22 Hz and three different main vibration frequencies (MVF) of 150 Hz, 225 Hz, and 300 Hz. The Type 3 signal was white Gaussian noise (WGN) with 0 dBW power.

**Figure 3:**
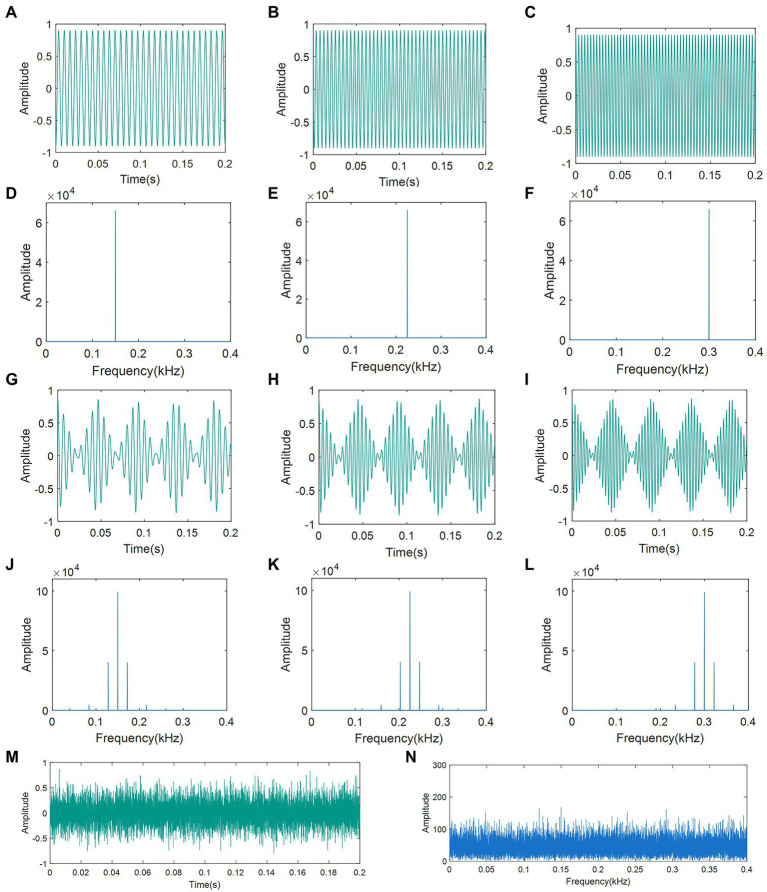
Oscillograms and spectrograms of artificial disruptive signals: **(A)** Oscillogram of PT (150 Hz); **(B)** Oscillogram of PT (225 Hz); **(C)** Oscillogram of PT (300 Hz); **(D)** Spectra of PT (150 Hz); **(E)** Spectra of PT (225 Hz); **(F)** Spectra of PT (300 Hz); **(G)** Oscillogram of CPS (22-150 Hz); **(H)** Oscillogram of CPS (22-225 Hz); **(I)** Oscillogram of CPS (22-300 Hz); **(J)** Spectra of CPS (22-150 Hz); **(K)** Spectra of CPS (22-225 Hz); **(L)** Spectra of CPS (22-300 Hz); **(M)** Oscillogram of WGN; and **(N)** Spectra of WGN. PT: pure tone; CPS: continuous pulse signal; WGN: white Gaussian noise.

All disruptive signals were synthesized using the Python NumPy library (NumPy, 2002), in which the CPS was obtained by filling a standard sine wave with a triangular pulse at a frequency of 22 Hz. The oscillograms and spectrograms of the seven artificial disruptive signals are presented in Figure 3.

### Playback of artificial disruptive signals

The experimental conditions were the same as the BPH rearing conditions. Healthy and insect-free rice seedlings were selected and pruned according to the experimental requirements. Well-developed male BPHs that had not mated after emergence for 2–3 days were placed into the circular observation box. Next, the box and BPH were left alone for more than 3 h. The playback device was placed at a height of 2 cm above the box and the signals were played back using a Sony recording device (model ICD-UX575F). To avoid the influence of intensity difference in the courtship and disruptive signals on BPH courtship responses, the pre-recorded courtship signal, and disruptive signal were mixed according to a uniform intensity.

To ensure that each tested male BPH was active, a female courtship signal was played to each BPH. If the tested BPH replied to the played signal within 10 s twice in a row, it was considered to be in the active reply period (ARP), which means that the tested BPH reacted strongly to the courtship signal and sent signals in response. Experiments were carried out on BPH that was in the ARP, and a combination of two control tests (courtship signal) and two disruptive tests (mixed-signal involving the courtship and disruptive signals) was used to test each disruptive signal in a loop. Each signal was played one syllable at a time by referring to segments of the courtship signals of BPHs. The responses of the BPHs were observed within 45 s after playback. If the BPHs replied, the delay of the reply was recorded. The main purpose of recording whether the males replied was to establish whether males could distinguish female courtship signals mixed with various kinds of disruptive signals. The reply delay was the time it took for the males to recognize and reply to the courtship signal.

After each signal was played back, we waited 60 s to confirm whether the tested BPH replied. If it did not reply, we waited a further 60 s before conducting the next test. If there was still no reply after the courtship signal was played a second time, we performed an additional test of the courtship signal. If the tested BPH still did not reply, we concluded that the tested BPH was temporarily not in the ARP, and this data was not recorded in the experimental results. Otherwise, the experiments continued. Besides, various behaviors of the tested BPHs, including movement, searching, localization, etc., were observed and recorded throughout the experiments. Each group of playback experiments is independent of the other, and the BPH subjects were discarded at the end of the experiment, meaning the number of tests per set was equal to the number of subjects.

### Evaluation of disruption effect on courtship behavior

To test whether the disruptive signals had a significant effect on the replies of males, a nonparametric test was performed on the data of reply delays. Besides, pairwise tests were conducted for paired data. Reply delays for the disruption-free signals and the seven kinds of artificially synthesized disruptive signals were compared using the Kruskal–Wallis test followed by Dunn’s pairwise comparison test (Kruskal and Wallis, 1952; Dunn, 1964). In this study, the statistical analysis was conducted using the Kruskal–Wallis H test in SPSS Statistics.

## Results and analysis

### Spectra of BPH courtship vibration signals

**Figure 4:**
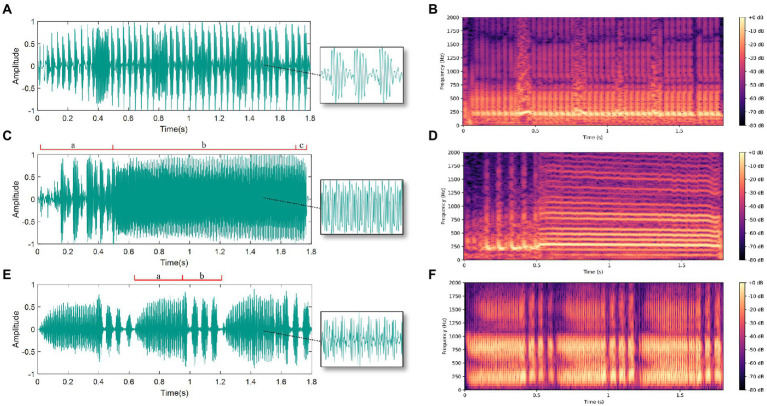
Oscillograms and spectrograms of male and female vibration signals: **(A)** Oscillogram of female courtship signal; **(B)** Spectrogram of female courtship signal; **(C)** Oscillogram of male courtship signal (divided into parts a, b and c); **(D)** Spectrogram of male courtship signal; **(E)** Oscillogram of male competition signal (divided into parts a and b); **(F)** Spectrogram of male competition signal.

In the experiment, we recorded 60 female courtship signals, 66 male courtship signals, and 39 male competition signals. Analysis of the BPH courtship signals indicates that the female signals exhibit a continuous pulse signal. Besides, by using the fast Fourier transform to analyze the frequency spectrum of the signal, we determined that its main vibration frequency is 236 ± 43 Hz and the pulse rate is 23 ± 2 Hz. The male signal is more complex and can be divided into a courtship signal and a competition signal. The courtship signal of males can generally be divided into three parts. The first part is three to ten irregular pulses (a), followed by a continuous pulse signal with a main vibration frequency of 255 ± 24 Hz (b), while the third part is zero to five wide pulses (c). The male BPH competition signal consists of continuous pulses (a) and then two to four short pulses (b), and the main frequency of the continuous pulse segment is 281 ± 46 Hz. The oscillograms and spectrograms of the three signals are displayed in Figure 4.

### Courtship disruption effect of disruptive signals

Playback experiments with disruption-free signals and seven kinds of artificially synthesized disruptive signals were carried out. The results of the reply rate and reply delay time are shown in Table 1.

**Table 1:** Screening results of BPH courtship-specific disruptive signals.

Disruptive signals		No. of tests	No. of replies	Reply rate	Mean (±SD) reply delay (s)
Control	None	45	43	95.6%	5.3 ± 3.3
Artificial Disruptive Signals	PT (150 Hz)	39	22	56.4%	8.0 ± 5.2
	PT (225 Hz)	42	17	33.3%	9.1 ± 5.6
	PT (300 Hz)	42	18	42.9%	9.3 ± 5.9
	CPS (PR: 22 Hz; MVF: 150 Hz)	42	28	66.7%	5.8 ± 3.2
	CPS (PR: 22 Hz; MVF: 225 Hz)	40	21	52.5%	7.2 ± 5.0
	CPS (PR: 22 Hz; MVF: 300 Hz)	40	22	55.0%	7.4 ± 6.0
	WGN	43	36	83.7%	5.0 ± 3.1

PT, pure tone; CPS, continuous pulse signal; WGN, white Gaussian noise; PR, pulse rate; and MVF, main vibration frequency.

**Figure 5:**
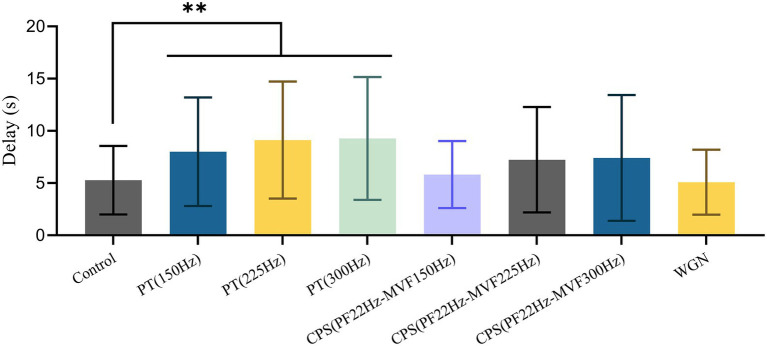
Mean ± SD of male reply delay from the female courtship signal with or without disruptive signals. Differences from the Kruskal–Wallis test followed by Dunn’s pairwise comparisons are denoted as follows: ^**^ *p* < 0.01.

Table 1 reveals that the male BPHs replied to female courtship signals in 95.6% of the control experiments. Besides, using Gaussian white noise resulted in a reply rate that was close to the control group, at 83.7%. In contrast, under the pure tone disruptive signal of 225 Hz, the reply rate of males was only 33.3%. The reply rate obtained with other disruptive signals ranged from 42.9 to 66.7%. As illustrated in Figure 5, there were significant differences in reply delay for BPHs that replied (Kruskal–Wallis test, *H* = 19.550, df = 7, *p* = 0.007^**^). Also, the results of the pairwise comparisons showed significant differences between the three groups of PT experiments and the control experiments (Dunn’s pairwise comparison test, Pcontrol-150 Hz = 0.009^**^, Pcontrol-225 Hz = 0.007^**^, Pcontrol-300 Hz = 0.005^**^). However, there were no significant differences for the other disruptive signals. The disruptive signals caused a substantial decrease in the reply rate of the tested BPHs and a considerable increase in the reply delay of the BPHs that replied. This indicates that playing the disruptive signal during the male recognition process of female courtship signals effectively disrupts the replies and localization of males, with a disruption rate of more than 50%. Also, the males that reply are less efficient in courtship due to the influence of the disruptive signals. Among the various disruptive signals tested, the disruptive effect of PT (225 Hz) was the best, followed by PT (300 Hz). The other synthetic disruptive signals also had a certain disruptive influence, while the WGN signal had almost no effect. Therefore, we presume that the BPH courtship signal recognition process is particularly sensitive to frequency. In cases when the CPS has the same intensity as the PT, the width of the CPS spectrum is greater. This means that the intensity of the CPS at the same frequency point is slightly weaker and the disruptive effect is lower than PT.

### Behavioral response

In the control experiments, the tested male BPHs exhibited stimulation after receiving the female courtship signals. There were two general modes of the tested BPHs in the behavioral observations. In Mode 1, the BPHs searched and localized during playback of the courtship signal and replied soon after the signal stopped. In Mode 2, the BPHs did not exhibit obvious searching or localizing behavior during the playback of the courtship signal, but they still replied to the playback signal after the signal stopped. In a large number of experiments, both modes were presented and the behavioral mode of a single tested BPH switched from time to time.

In the experiments using disruptive signals, the aroused behavior of the tested BPHs was lower than in the control experiments. There was no reply to the playback signal from 62.2% of the tested BPHs, and behavioral observations indicated that the tested BPHs did not show any obvious movement during playback or after playback stopped. The BPHs that replied were mostly of the second behavioral mode mentioned above, meaning that active searching and locating behavior was very rare. These observations demonstrated that the disruptive signals had a significant effect on the courtship behavior of BPHs.

## Discussion

The rice pest BPH does not have sex pheromones. Instead, it vibrates its abdomen at specific frequencies for courtship, localization, and eventually mating and egg-laying (Zhang and Chen, 1987a). To date, chemical control is still the primary method used to regulate the population of BPHs. This has caused increased BPH resistance to pesticides, rice pesticide residue, and serious environmental pollution (Wu, 2018). Thus, it is beneficial to use disruptive signals that upset the courtship of male and female BPHs, thereby delaying mating and reducing the mating rate. The bioacoustic method for disrupting the mating of *S. titanus* is currently under development and it has presented proof of concept under small-scale field conditions (Polajnar et al., 2016). While current studies on the vibration control of BPHs are limited to the disruptive effect of male competition signals (Fu et al., 1997, 1999), this paper aims to extract more effective disruptive signals for disturbing courtship responses.

Currently, costly equipment such as the laser vibrometer is commonly used in vibration studies of *S. titanus* (Mazzoni et al., 2009). In this paper, a system for collecting, monitoring, and playback of BPH vibration signals was developed using magnetoelectric converters, low-noise amplifier circuits, transducers, and other components. The system we devised achieved higher functionality with lower costs. Thus, it has the potential to be applied to more species of planthoppers and leafhoppers, thereby facilitating more insect vibration studies.

Vibration courtship disruption involves the use of specific disruptive signals to precisely upset the communication of BPHs which are in the courtship stage. It blocks or disrupts the normal courtship behavior between males and females and effectively prevents or controls overall mating behavior. BPH courtship signals stimulate sexual arousal in the opposite sex and are so specific that BPHs are insensitive to other types of signals. Therefore, the development of disruptive signals using the characteristics of courtship signals may affect the perception process of courtship signals. This is different from the method devised by Fu et al. (1997, 1999), which utilized male competition signals. The disruption method of the competition signal does have some influence, but the mutually competitive behavior of males is quite extensive in the BPH population and the disruption effect is limited. In contrast, the specific disruptive signal obtained through screening emphasizes the perception process. Therefore, it has better potential and wider applicability. Furthermore, analysis results of male and female courtship signals show that although the signals differ greatly in the time domain, the frequency range of the main vibrations is relatively stable. Therefore, normal courtship communication can be disrupted by playing back disruptive signals in the same frequency range during the courtship process between male and female BPHs. However, the BPH courtship and localization process is very conservative, with males generally initiating calls and then locating the female based on the female’s response (Zhang, 1997). Also, the male courtship signals are slightly more complex in the time domain, with different main vibration frequencies for male and female courtship signals. Therefore, we focused on the male recognition process of female signals to design experiments that screened disruptive signals for upsetting the courtship process of BPH males and females.

By using artificially synthesized disruptive signals with different frequency combinations for playback and monitoring experiments, a pure tone signal of 225 Hz was extracted. This signal affected male BPH recognition of the female’s courtship call. Experiments revealed that the male BPH reply rate decreased from the original 95.6 to 33.3%. Furthermore, the mean reply delay increased from 5.3 s to 9.1 s. This confirmed that the mean reply delay of males under disruption was significantly different from the control group. Therefore, it is feasible to use specific disruptive signals to disrupt the courtship behavior of BPHs. We hypothesize that the effectiveness of the 225 Hz pure tone is due to its disruption of the spectral structure of the female courtship signal, making it difficult for males to recognize or become sexually aroused.

By applying the mating disruption technique for *S. titanus*, the mating inhibition rate was about 90% (Polajnar et al., 2016). Due to its satisfactory experimental effects, this method has tangible potential for controlling *S. titanus* reproduction. The experiments in this paper only focused on behavior at the courtship stage, but our results confirmed the hypothesis that disruption with specific disruptive signals efficiently disturbs BPH courtship behavior. Furthermore, our results provide support for subsequent research on BPH control methods.

Admittedly, there are limitations to the findings of our study. The effects of the duration and frequency of the disruptive signals on courtship behavior, as well as on mating and egg-laying, still need to be tested experimentally. Additionally, the experiments in this paper were conducted indoors. However, if the results are applied to paddy fields, there will be a great deal of external interference noise, such as the shaking of the rice plants due to wind, noise from the surrounding environment, etc. For *S. titanus*, standards in viticulture provide delivery means of vibrational energy to target surfaces (Polajnar et al., 2016). However, for BPHs, the choice of delivery medium is still an issue. If the experimental technique is applied to paddy fields, substantial research efforts are still required to determine what equipment and what medium can be used to effectively deliver disruptive signals to BPHs on rice stems. In the next stage of our research, we will observe the effect of simulated external noise on BPH courtship and further investigate the effect of disruptive signals on BPH mating. Moreover, we will enhance the playback equipment to address certain issues such as avoiding environmental pollution, reducing costs, and expanding the effective coverage range. These will be very challenging but interesting tasks.

## Data availability statement

The raw data supporting the conclusions of this article will be made available by the authors, without undue reservation.

## Author contributions

ZF, ZY, and QY came up with the idea and wrote and revised the manuscript. ZF, QW, and BY purposed the experimental methods. YG, JL, YD, and JB analyzed the experimental data. All authors contributed to the article and approved the submitted version.

## Funding

This study is supported by the Key R&D Program of Zhejiang (No. 2022C02004).

## Conflict of interest

The authors declare that the research was conducted in the absence of any commercial or financial relationships that could be construed as a potential conflict of interest.

## Publisher’s note

All claims expressed in this article are solely those of the authors and do not necessarily represent those of their affiliated organizations, or those of the publisher, the editors and the reviewers. Any product that may be evaluated in this article, or claim that may be made by its manufacturer, is not guaranteed or endorsed by the publisher.
